# Loss of p27^kip1^ increases genomic instability and induces radio-resistance in luminal breast cancer cells

**DOI:** 10.1038/s41598-017-00734-3

**Published:** 2017-04-04

**Authors:** Stefania Berton, Martina Cusan, Ilenia Segatto, Francesca Citron, Sara D’Andrea, Sara Benevol, Michele Avanzo, Alessandra Dall’Acqua, Monica Schiappacassi, Robert G. Bristow, Barbara Belletti, Gustavo Baldassarre

**Affiliations:** 1 Division of Molecular Oncology, CRO of Aviano, National Cancer Institute, Aviano, 33081 Italy; 2 Princess Margaret Cancer Center, University Health Network, Toronto, Ontario Canada; 3 Division of Medical Physics, CRO of Aviano, National Cancer Institute, Aviano, 33081 Italy; 4 Department of Medical Biophysics, University of Toronto, Toronto, Ontario Canada

## Abstract

Genomic instability represents a typical feature of aggressive cancers. Normal cells have evolved intricate responses to preserve genomic integrity in response to stress, such as DNA damage induced by γ-irradiation. Cyclin-dependent kinases (CDKs) take crucial part to these safeguard mechanisms, but involvement of CDK-inhibitors, such as p27^Kip1^, is less clear. We generated immortalized fibroblasts from p27^kip1^ knock-out (KO) mouse embryos and re-expressed p27^kip1^ WT, or its mutant forms, to identify the function of different domains. We γ-irradiated fibroblasts and observed that loss of p27^Kip1^ was associated to accumulation of residual DNA damage, increased number of mitotic aberration and, eventually, to survival advantage. Nuclear localization and cyclin/CDK-binding of p27^Kip1^ were critical to mediate proper response to DNA damage. In human luminal breast cancer (LBC) p27^kip1^ is frequently down-modulated and CDKN1B, p27^Kip1^ gene, sporadically mutated. We recapitulated results obtained in mouse fibroblasts in a LBC cell line genetically manipulated to be KO for CDKN1B gene. Following γ-irradiation, we confirmed that p27^kip1^ expression was necessary to preserve genomic integrity and to recognize and clear-out aberrant cells. Our study provides important insights into mechanisms underlying radio-resistance and unveils the possibility for novel treatment options exploiting DNA repair defects in LBC.

## Introduction

The maintenance of genomic integrity is a fundamental need in cell biology. Given the potentially devastating effects of genomic instability, cells have developed a complex series of mechanisms to preserve their genetic heritage^1^. Besides the mechanisms directly involved in preventing and sensing the DNA damage, cells control genomic integrity by activating and coordinating the so-called DNA damage response, responsible for activation of cell cycle checkpoints and, when necessary, of programmed cell death, in order to delay and/or avoid proliferation of damaged cells, with consequent propagation of genetic defects^2, 3^.

The tumor suppressor p27^Kip1^ (hereafter called p27) has been originally identified as a cyclin-dependent kinase (CDK) inhibitor, being able to bind and restrain the activity of virtually all cyclin-CDK complexes. p27 also displays CDK-independent activities, including the participation to the DNA damage response^4, 5^. Previous data suggested that p27 accumulation, due to the inhibition of the ubiquitin ligase Skp2, is necessary for a proper response to DNA damage^6–8^. Interestingly, studies from mouse models suggest that accumulation of p27 in G2 (due to Skp2 knock-out) has profound effect on proliferation, cell size and DNA content.

Nevertheless, p27 knock-out (p27KO) mice and primary mouse embryo fibroblasts (MEF) are highly sensitive to genotoxic stress and, in particular, to radiation (IR)^7, 9^. Following low doses of ionizing radiation p27KO cells showed impaired G2/M arrest coupled with a higher number of chromatid breaks and micronuclei formation if compared to wild type (WT) cells^7^. In particular p27 deficiency resulted in a defect in the early radiation-induced G2/M arrest, suggesting a physiologic role for p27 protein in the immediate response to genotoxic insult^7^.

Following cell irradiation, the G2/M checkpoint is quickly activated to prevent that damaged DNA is inherited by daughters cells but a threshold of DNA damage exists, both for the activation and the resolution of the checkpoint^10^. G2/M checkpoint activation and resolution relay on the inhibition of CDK1 activity and it has been calculated that occur when cells harbor 10 to 20 unrepaired DNA double strand breaks (DSB)^10^. As a consequence low doses of radiation, resulting in low number of DSB, fail to completely prevent the entry in mitosis of damaged cells^10^.

The role of p27 in response to radiation has been only limitedly studied in mouse models and very little is known regarding the effects of p27 loss following low doses of radiation in human cells.

Recent whole genome sequencing data suggest that CDKN1B (the gene encoding for p27) is frequently mutated in some types of human cancer, particularly in luminal breast cancer^11–13^. Mutations of CDKN1B in luminal breast cancer occur, in more than half of the cases, in the C-terminal portion of the protein, suggesting that tumor suppressive activities are present in this region^11–13^.

For an optimal local control of the disease, locally advanced luminal breast cancers are usually treated with wide local excision, followed by radiotherapy^14^. In light of the evidences reported above, we decided to investigate if p27 expression and/or mutation affected the response to radiation, possibly driving disease relapse, by comparing well controlled human and mouse systems.

Here, we addressed these points by generating and characterizing mouse and human p27KO and knock-in (KI) cells and dissecting the role of different p27 domains in the control of DNA damage response induced by ionizing radiations. We highlight an important correlation between loss of p27 and radio-resistance of luminal breast cancer cells that could eventually result in breast cancer relapse in patients.

## Results

### Generation and characterization of 3T3 fibroblasts expressing different p27 mutants

**Figure 1:**
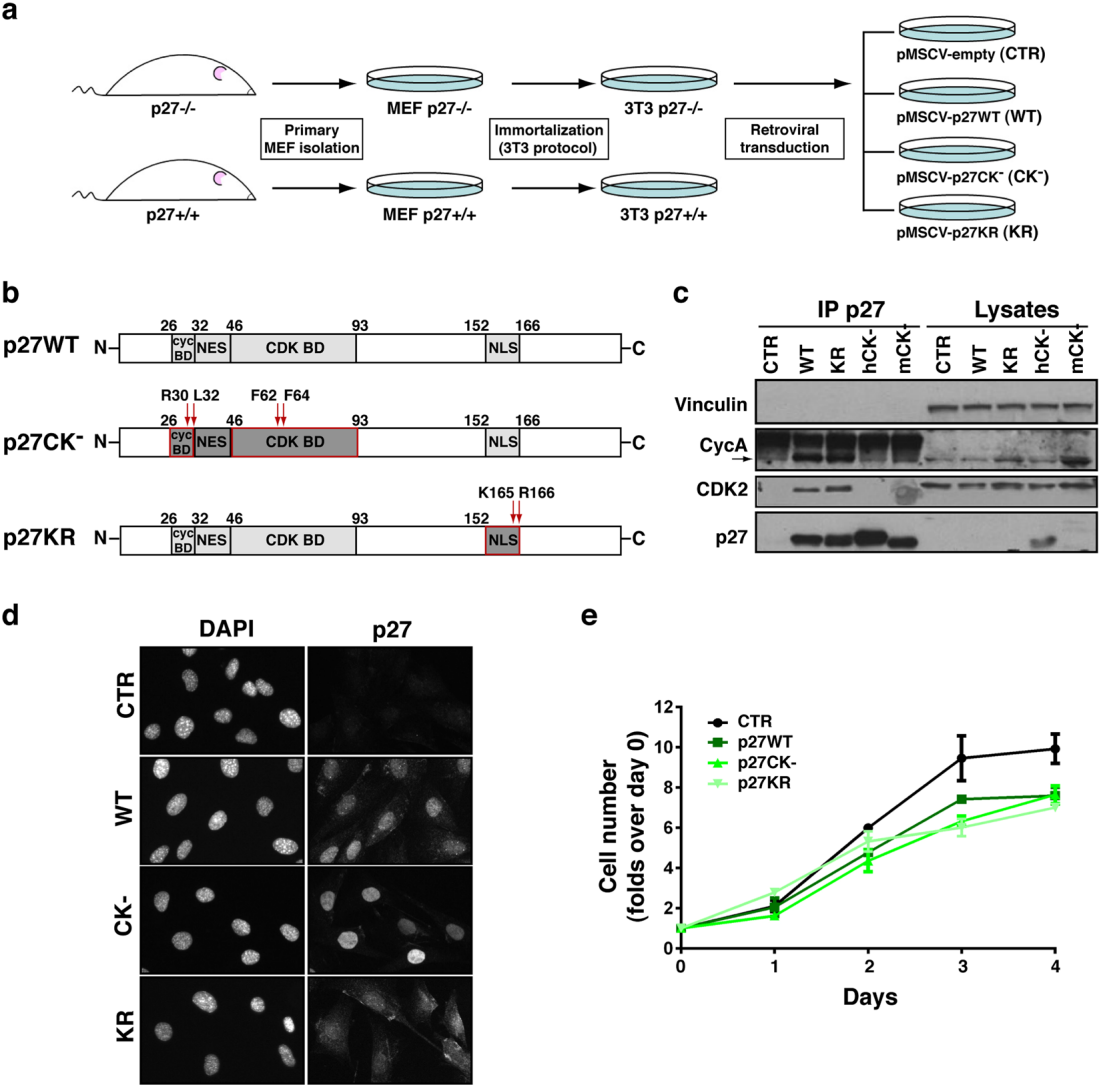
Generation of p27KO and mutant p27 3T3 mouse fibroblasts. (**a**) Schematic representation of the experimental workflow used to generate 3T3 p27 mutant clones. Briefly, fibroblasts were harvested from p27KO mouse embryos, immortalized using the 3T3 protocol and transduced with p27^WT^ or mutants of interest. (**b**) Schematic representation of p27^WT^ protein structure and the two mutants of interest, generated by point mutations in the cyclin- CDK- binding domains (p27^CK−^) or in the nuclear localization signal (p27^KR^). (**c**) Western Blot analysis of immunoprecipitated (IP) proteins and lysates harvested from indicated 3T3 cell clones. CTR represents p27KO fibroblasts; WT, KR, CK− represent respectively the p27WT, KR and CK− represents (both human and mouse) transduced-fibroblasts. (**d**) Immunofluorescence analysis of above described clones, showing p27 expression and localization. Nuclei were stained with DAPI. (**e**) Growth curve analysis of above described clones. Cells were plated in 6-well plates in duplicates and counted every day, for 4 consecutive days. Data are expressed as fold increase over the number of plated cells (1 × 10^5^) with standard deviation. MEF, mouse embryo fibroblasts; BD, binding domain; NLS, nuclear localization signal; NES, nuclear export signal.

We used mouse fibroblast cell cultures derived from p27 *wild type* (p27WT) and p27 *knock out* (p27KO) mouse embryos, immortalized by 3T3 protocol^15, 16^. The p27KO cell culture has been also used to stably express different forms of p27 cDNAs (Fig. 1a). We re-expressed p27 in the wild type form (p27^WT^) or in two mutant forms, the CK− (p27^CK−^) and the KR (p27^KR^) (Fig. 1b). The CK− mutant does not bind cyclins or CDK, carrying point mutations that affect both the Cyclin and the CDK binding domains (R30A-L32A-F62A-F64A)^16–18^. The KR variant carries point mutations in the nuclear localization signal (K165A-R166A), with a consequent retention mainly into the cytoplasm^16, 19^. The p27^CK−^ mutant is usually expressed at higher levels respect to p27^WT^ protein since it is not subjected to proteasome-dependent degradation and is, therefore, more stable in proliferating cells^16, 20^. Accordingly, in exponentially growing conditions, the p27^CK−^ mutant was expressed at higher levels compared to the WT and KR forms, both in western blot and immunofluorescence analyses (Fig. 1c,d). As expected, p27^WT^ was expressed both in the nucleus and in the cytoplasm, whereas p27^KR^ was predominantly localized in the cytoplasm and p27^CK−^ in the nucleus (Fig. 1d). The association of p27 with cyclins (*e*.*g*. as cyclin A) and CDKs (*e*.*g*. as CDK2) was abrogated when the CK− form was expressed into the cells, whereas the association was confirmed in the case of p27^WT^ and KR mutant (Fig. 1c). Compared to the p27KO cells, used as control (expressing the empty vector pMSCV-PAC), the expression of p27 (either WT or mutant) was associated with a slight decrease in their proliferative rate (Fig. 1e).

### Defective p27 expression is associated to higher residual DNA damage after IR

Literature data showed a role of p27 in protecting from mutations, chromosome damage and centrosome amplifications generated by DNA damaging agents, whereas p27 deficiency leads to an increase of chromosomal aberrations^6–8^.

**Figure 2:**
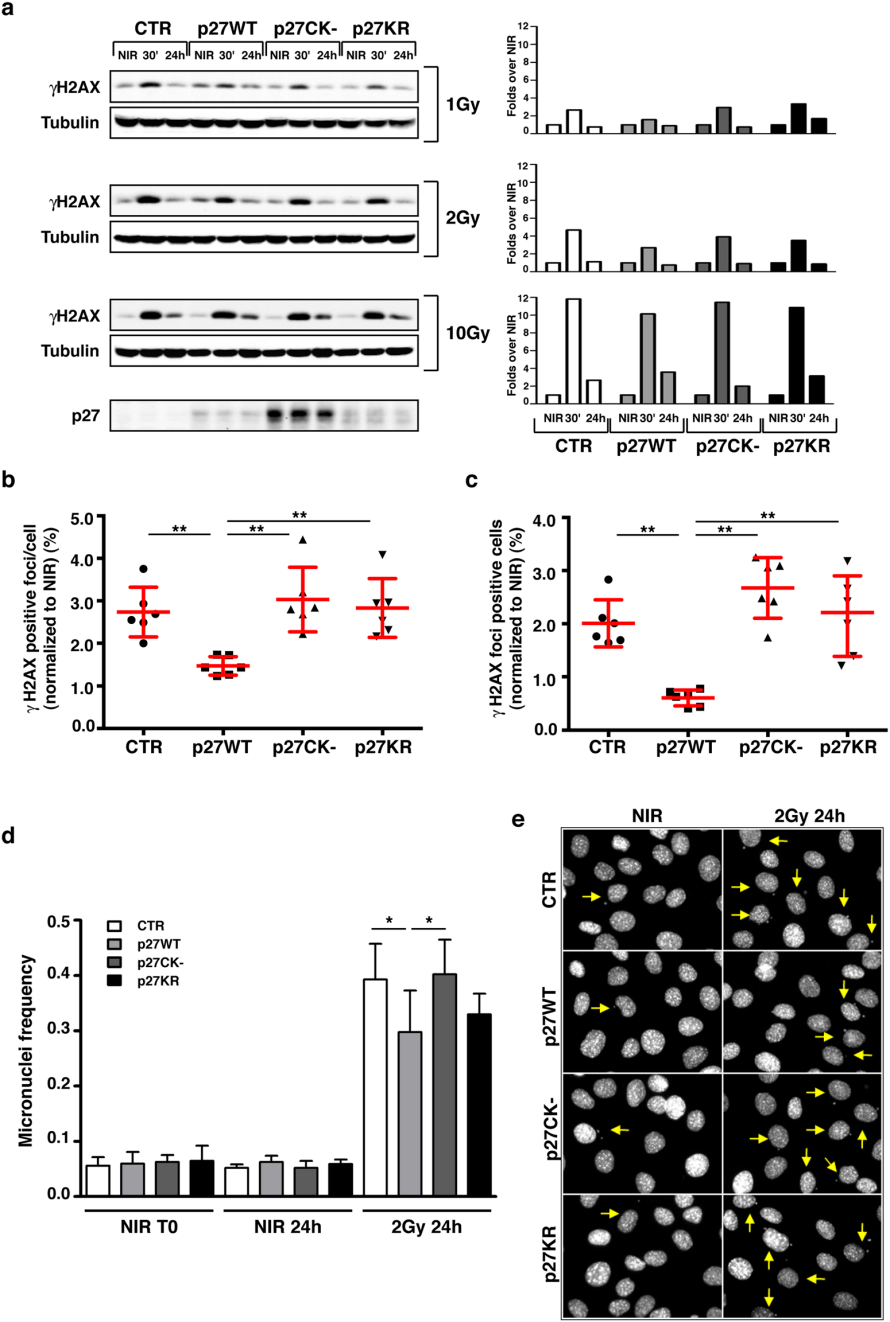
IR of p27KO cells is associated to high residual DNA damage and micronuclei frequency. (**a**) Western Blot analysis of phospho-Ser139-Histone 2AX (γH2AX) in lysates from indicated cell clones, non-irradiated (NIR) or irradiated with 1, 2 and 10 Gy and harvested after 30 minutes or 24 hours. Tubulin was used as loading control. The bottom panel shows p27 expression levels in the different cell clones. On the right, quantification of normalized levels of γH2AX is reported. (**b**,**c**) Graphs report the quantification of the γH2AX positive foci/cell (**b**) and the γH2AX positive cells (**c**), at 24 hours after 2 Gy, normalized for the number of the corresponding NIR and expressed as mean of three independent experiments performed in duplicates, with SD. (**d**) Graph reports the frequency of micronuclei positivity found at 24 hours in NIR cells and 2 Gy irradiated cells expressed as percentage of positive cells over the total cell number. (**e**) Representative pictures of NIR cells (left panels) or 2 Gy irradiated cells after 24 hours (right panels), showing the presence of micronuclei (yellow arrows). Cells were stained with DAPI. *Indicates a p ≤ 0.05; **p ≤ 0.01.

To investigate the role of p27 in the response to DNA damage and the involvement of its different protein domains, we analyzed the response after γ-irradiation of p27deficient (p27^KO^, CTR) and p27-rescued (p27^WT^, p27^CK−^ and p27^KR^) mouse fibroblasts. Previous data demonstrated that p27 plays a minor role in the DNA damage response activated after high single dose of radiation (10 Gy and higher)^7^. To highlight a possible impact of p27 in the control of G2/M block induced by IR we thus decided to focus our characterization in low-intermediate radiation doses, using mainly 1 Gy and 2 Gy. Moreover, a dose of 2 Gy more likely represents a clinically relevant dose in cancer therapy, frequently used in the hypo-fractionated radiotherapy regimens^21, 22^. As measure of chromosomal damage, we first analyzed the expression of the phosphorylated form of histone H2AX (Ser139-H2AX, i.e. γH2AX), a very sensitive and well-established marker for DNA double-strand breaks (DSBs). H2AX becomes phosphorylated around DSBs within minutes after exposure to ionizing radiation (γH2AX foci), thus marking the site for assembly of numerous repair and cell cycle-regulating proteins. At early time points (30 minutes) and low-intermediate doses of irradiation (1–2 Gy), p27 deficient cells displayed a slightly higher activation of this signaling compared to p27^WT^ cells (Fig. 2a), supporting the literature data collected using MEFs^7^. Weak differences were detectable after high (10 Gy) radiation doses or at late time points (24 hours) (Fig. 2a). Phosphorylation of H2AX occurs immediately after the formation of a DSB, and could be observed in cells in active phase of DNA duplication (S-phase of the cell cycle) or those in apoptosis^23^. Since p27KO cells had a higher proliferation rate (Fig. 1e and refs 16, 24) to reduce the bias related to those DSBs not directly induced by IR, we analyzed the rate of positive γH2AX foci by immunofluorescence in EdU negative cells (*i*.*e*. cells not in S phase) 24 hours after IR. In this condition, we found a significant increase in the residual γH2AX foci in p27 null fibroblasts compared to those expressing p27 WT, in terms both of number of foci/cell and number of H2AX positive cells (Fig. 2b and c, respectively). In this setting, cells expressing the mutant forms of p27, CK− and KR, displayed the same behavior of p27 null cells. The expression of p27 was retained after IR, both at early (30 minutes) and late (24 hours) time points, without remarkable alterations (Supplementary Fig. S1). These data suggest that the nuclear localization and the binding to cyclin-CDKs are important requisites for an efficient repair of IR-induced DNA damages.

### p27KO cells display increased micronuclei frequency and mitotic aberrations

Micronuclei (MN) frequency, a marker of unsolved DNA damage, is very low in normal proliferating cells and is widely used as a marker for evaluating cell damage after radiation or chemical exposure^25^.

MN frequency did not change in non irradiated and dividing cells among the different cell lines, being on average around 6% in all of them (CTR, 5.35%; p27^WT^, 6.05%; p27^CK−^, 5.65%; p27^KR^, 6.15%) (Fig. 2d). Yet, 24 hours after ionizing radiations (2 Gy) the re-expression of p27^WT^ was associated with a significant reduction in the number of cells carrying micronuclei, when compared to p27KO and p27^CK−^ cells while cells expressing p27^KR^ displayed an intermediate phenotype (Fig. 2d and e).

**Figure 3:**
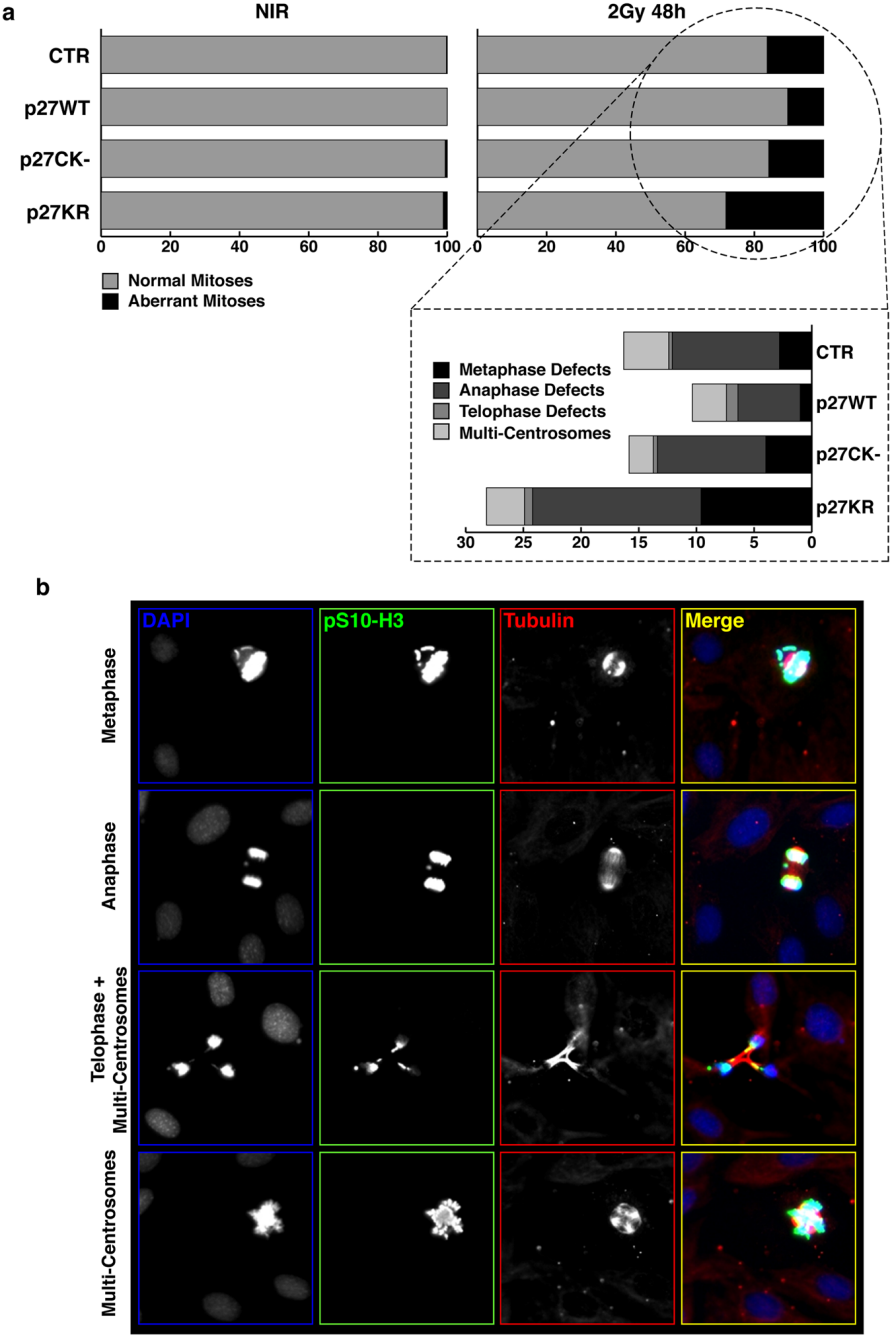
IR of p27KO cells is associated to mitotic aberrations. (**a**) Graph reports the quantification of aberrant mitoses encountered in the different cell clones when non-irradiated (NIR, left panels) or irradiated with 2 Gy (right panels) at 48 hours after radiation. Data are expressed as % of total cells. At the bottom, the aberrations were sub-classified respect to mitotic phases. (**b**) Representative pictures of cells reporting the above mentioned mitotic aberrations. Cells were stained for phospho-Ser10 Histon-H3 (pS10-H3) to identify mitotic cells (pseudocolored in green, shown in white), αTubulin to highlight the mitotic spindle (pseudocolored in red, shown in white) and DAPI to identify the cell nuclei (pseudocolored in blue, shown in white). Only the merged picture is shown with colors.

Micronuclei often originate from unrepaired DSB and, always, depend on aberrant mitoses. Accordingly, unrepaired DSBs can be responsible for mitotic defects and are ultimate lesions for the formation of chromosomal aberrations. We thus analyzed p27KO and knockin cells for the presence of mitotic defects. Cells were irradiated with 2 Gy and, after 48 hours, fixed and stained for phospho-Ser10 Histone H3 (marker of mitotic cells) and α-tubulin (marker of the mitotic spindle). In non irradiated cells, the number of aberrant mitoses was extremely low and, as observed for micronuclei frequency, no remarkable difference was associated with the presence/absence of p27 (Fig. 3a). After irradiation, the re-expression of p27^WT^ was associated with the lowest number of mitotic defects, in comparison to the other cell clones (Fig. 3a). As exemplified in Fig. 3b, we could not highlight a remarkably different pattern in the type of aberrant mitoses scored between the cell clones, suggesting that the high number of mitotic defects was due to higher genetic instability of these cells after ionizing radiations.

### Loss of p27 leads to survival advantage after genotoxic stimuli

**Figure 4:**
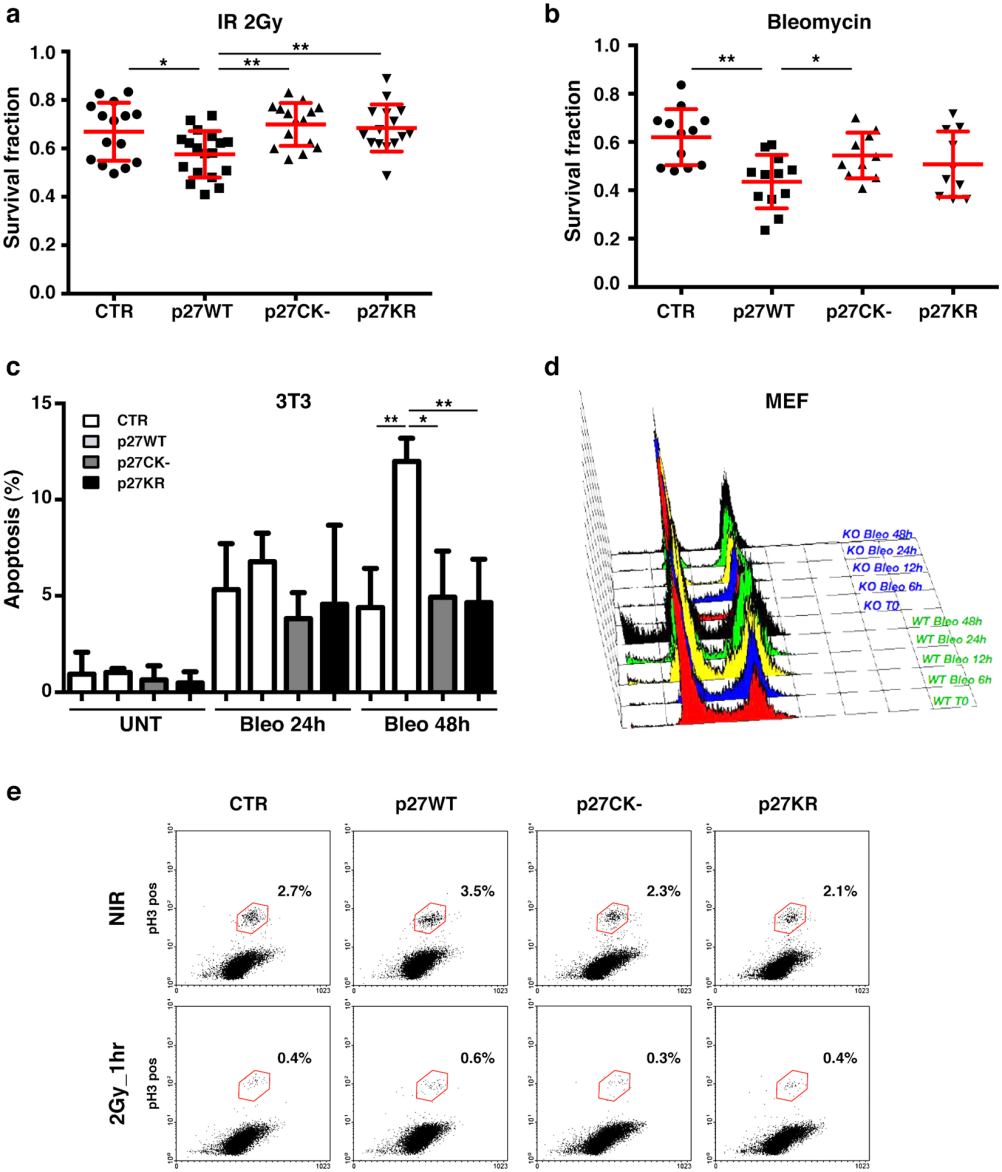
p27KO cells display survival advantage after genotoxic stimuli. (**a**) Graph reports the survival fraction of the different cell clones subjected to 2 Gy IR and then plated in clonogenic assay. Data are expressed as mean of three independent experiments performed at least in triplicates, with SD. (**b**) Graph reports the survival fraction of the different cell clones subjected to the radiomimetic agent Bleomycin (6.25 µg/ml for 2 hours) and then plated in clonogenic survival assay. Data are expressed as mean of three independent experiments performed at least in triplicates, with SD. (**c**) Graph reports the percent of apoptotic 3T3 p27KO (used as control = CTR) and mutant clones, after treatment with Bleomycin (6.25 µg/ml for 2 hours) at 24 and 48 hours. Apoptotic cells were assessed by nuclear morphology and membrane integrity after DAPI staining. (**d**) Graph reports the cell cycle distribution, evaluated by FACS analysis, of mouse fibroblasts from WT and p27KO embryos (MEF), treated with Bleomycin (5 µg/ml for 2 hours) for the indicated time points. (**e**) Plots report the percent of pS10-H3 positive cells 1 hour after 2 Gy IR or NIR, evaluated by FACS, in 3T3 p27KO (CTR) and mutant clones, as indicated. *Indicates a p ≤ 0.05; **p ≤ 0.01.

Following IR, high levels of DNA damage and accumulation of aberrations are often associated with increased radio-sensitivity rather then -resistance^26^. To evaluate which was the case in p27KO cells, we irradiated and tested cells in clonogenic survival assays, after different doses of IR (1–2–6 Gy). In line with our previous results (Fig. 2) and with others’ findings^7^, no differences in cell survival after high doses of IR (6 Gy) were detectable. However, after 2 Gy, cells re-expressing p27WT displayed a significant and consistent decrease in survival respect to p27KO cells (Fig. 4a). The same result was obtained using the radiomimetic drug Bleomycin (Fig. 4b). After bleomycin treatment, we found a significant increase in the apoptotic rate of cells expressing p27WT, both in 3T3 fibroblasts and in primary mouse embryo fibroblasts (MEFs) (Fig. 4c and d).

To better analyze the phenotype of p27KO cells, we quantified the phosphorylation of S10-Histone H3 (pH3), a marker for mitotic cells. FACS analysis of pH3-positive cells, both at 1 hour (early G2 checkpoint) and 16 hours (late G2 checkpoint, data not shown) after IR, revealed that the different cell clones similarly activated the mitotic checkpoint, all displaying a drop in number of pH3-positive cells (Fig. 4e).

Overall, these data indicated that, following irradiation, the absence of p27 or the expression of the mutant forms p27^CK−^ and p27^KR^ were similarly associated with an increased survival fraction. However, the mitotic checkpoints were still correctly activated both in the presence and in the absence of p27.

### Generation and characterization of MCF-7 luminal breast cancer cells KO and KI for p27

We next asked whether our findings on mouse fibroblasts could have a clinical implication in breast cancer where p27 expression and localization have been frequently associated with prognosis and response to therapies^27, 28^. In particular, we focused on luminal breast cancer (LBC) that, as mentioned above, is the breast cancer subtype with the highest frequency of CDKN1B mutations^11–13^ and chose the MCF-7 cell line as a validated model of LBC.

**Figure 5:**
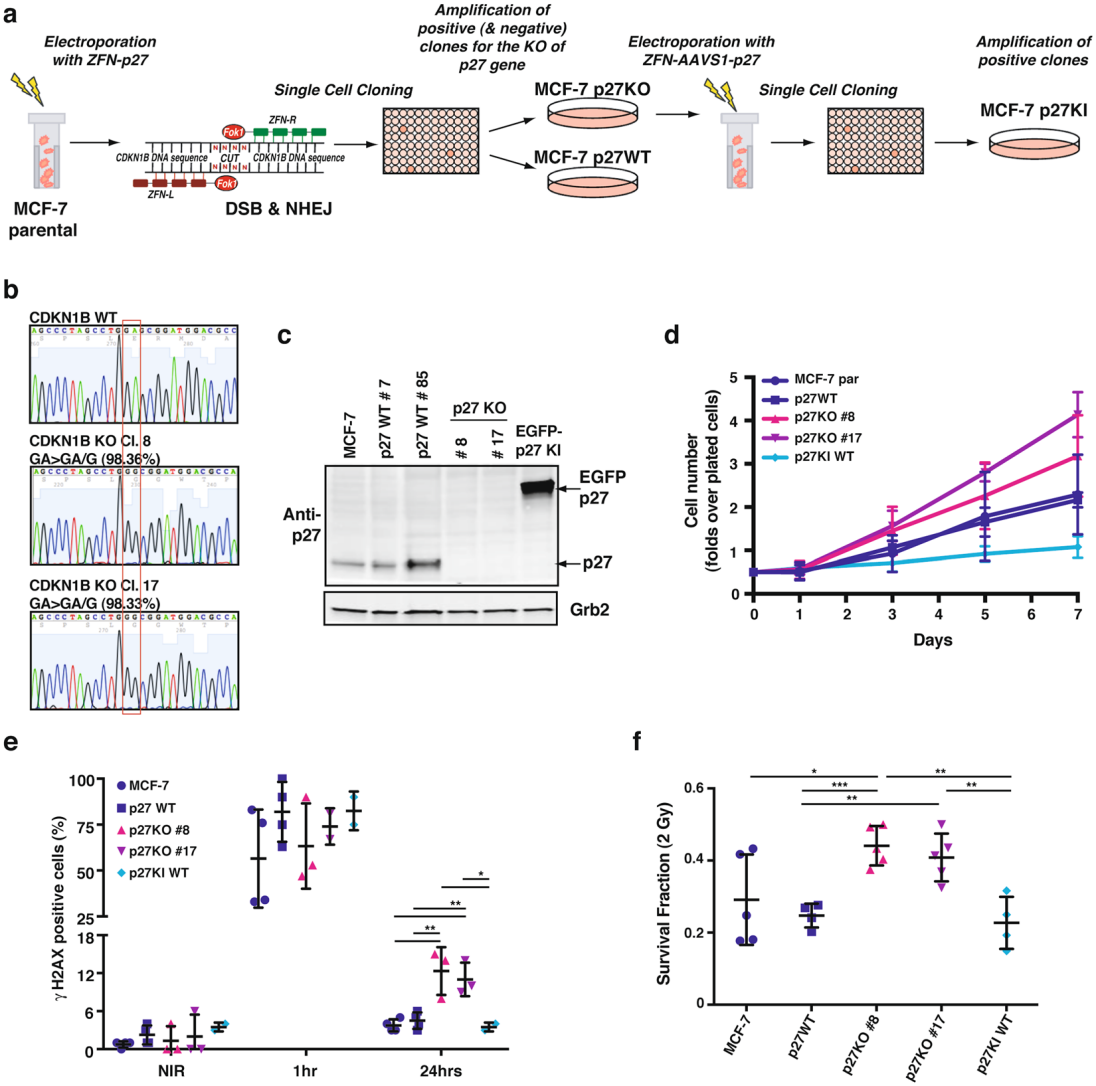
Generation of MCF-7 luminal breast cancer cells KO and KI for p27. (**a**) Schematic representation of the experimental workflow used to generate MCF-7 p27KO and KI clones. Briefly, MCF-7 cells were electroporated to incorporate vectors encoding for engineered ZFNs cutting at the CDKN1B gene. Consequent events of DSB and NHEJ were exploited to obtain p27KO clones. Following validation of CDKN1B gene KO, the identified clone was further electroporated with vectors encoding for engineered ZFNs, annealing on the AAVS1 safe harbor gene. The reaction was carried out in the presence of p27WT DNA sequence (fused to EGFP), to induce the generation of p27KI clones through integration. (**b**) Representative images of the DNA sequence, evaluated by Sanger method, corresponding to CDKN1B gene, showing the WT and the mutated sequences in two p27KO clones (#8 and #17). The two clones both display a single base deletion, confirmed also by NGS. (**c**) Western Blot analysis of p27 expression of two WT, two p27KO and 1 EGFP p27KI clone. Grb2 was used as loading control. (**d**) Growth curve analysis of above described clones. Cells were plated in 6-well plates and counted every other day, for 7 days. Data are expressed as fold increase over the number of plated cells (5 × 10^4^) and represent the mean of three independent experiments performed in duplicates, with SD. (**e**) Graph reports the quantification of the γH2AX positive cells in NIR cell clones at 1 hour and 24 hours after 2 Gy. (**f**) Graph reports the survival fraction of the different MCF-7 cell clones subjected to 2 Gy and then plated in clonogenic assay. ZFN, zinc fingers nucleases; DSB, double strand breaks; NHEJ, non-homologous end joining; AAVS1, Adeno-Associated Virus Integration Site 1; EGFP, enhanced green fluorescent protein. *Indicates a p < 0.05; **p < 0.01; ***p < 0.001.

We exploited the Zinc Finger Nucleases (ZFNs) technology to generate p27 Knock Out (KO) clones of MCF-7 cells (Fig. 5a). Loss of a nucleotide at the predicted cut site, in position 40 of CDKN1B exon 1, was found by Sanger sequencing in 25/25 cloned fragments and in >98% amplicons by NGS (Fig. 5b and data not shown) of selected clones. This nucleotide loss led to a frameshift and a stop codon formation, generating a truncation of the protein at aminoacid 15 and resulting in complete protein loss in the two selected p27KO clones (#8 and #17). To avoid bias due to clonal selection, at this stage we also generated two different types of controls: i) we isolated clones that were generated in parallel with the p27KO ones, but resulting completely negative for CDKN1B Knock Out (MCF-7 p27WT #7 and #85); ii) using one of the MCF-7 p27KO clones, we generated p27 Knock In (KI) clones, by targeted integration of EGFP-p27 WT in the AAVS1 safe harbor locus (MCF-7 p27KI WT). These cell clones were then used with MCF-7 parental cells (MCF-7) to better characterize the behavior of MCF-7 p27KO cells (Fig. 5a). Western blotting on either immunoprecipitated proteins or on whole lysates confirmed the absence of p27 protein in the KO clones and the expression of the EGFP-tagged protein in KI one (Fig. 5c and Supplementary Fig. S2A).

We first assessed whether loss of p27 affected the proliferative behavior of these cancer cells. In growth curve experiments, we could detect a reproducible, but not statistically significant, increase of cell number in p27KO clones with respect to the controls (Fig. 5d). On the other hand, the re-expression of p27 (MCF-7 p27KI WT) induced a strong inhibition of proliferation (Fig. 5d).

### p27KO luminal breast cancer cells accumulate residual DNA damage after IR

We next analyzed whether loss of p27 caused a different DNA damage response by irradiating cells with 2 Gy and then looking at γH2AX positive cells, after 1 and 24 hours from IR. In line with the results obtained in mouse fibroblasts, a significant increase in the residual γH2AX foci after 24 hrs from DNA damage was observed in p27KO clones, in terms of both number of γH2AX positive cells (Fig. 5e) and number of positive foci/cell (not shown). Next, clonogenic survival assay with 2 or 5 Gy demonstrated that, at the intermediate dose of 2 Gy, the survival fraction of p27KO cells was significantly higher than that of p27 expressing cell (Fig. 5f; Supplementary Fig. S2B).

### p27KO luminal breast cancer cells accumulate mitotic defects after IR

**Figure 6:**
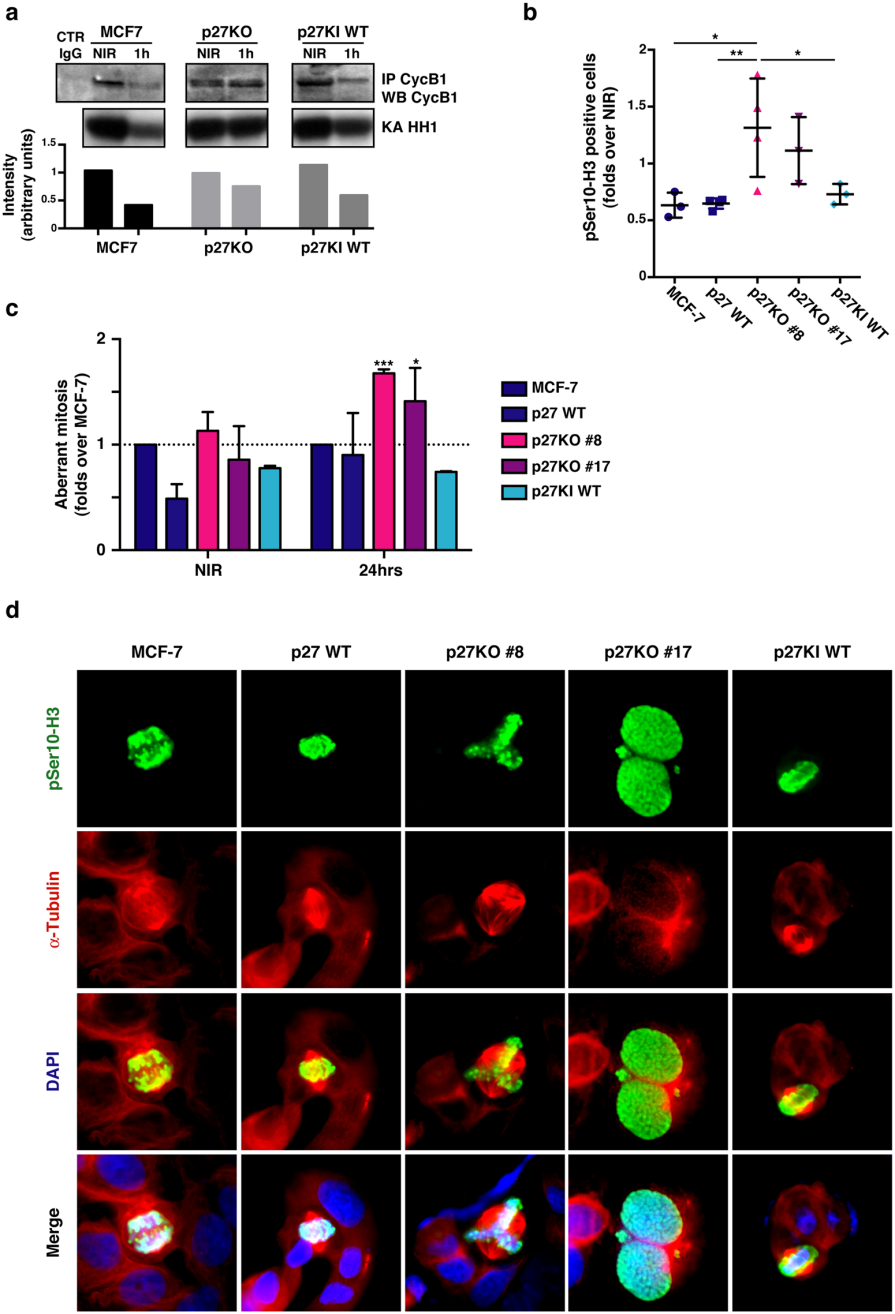
Luminal breast cancer cells KO for p27 are radio-resistant and accumulate mitotic defects. (**a**) Blots report the kinase assay (KA) on Histon H1 (HH1) by cyclin B1-associated CDK activity (lower panels). Upper panels show the level of immunoprecipitated cyclin B1 in the indicated cell clones. Bottom graph reports the quantification of HH1 phosphorylation, normalized for the quantity of immunoprecipitated cyclin B1. Full-length blot is shown in Supplementary Fig. S3. (**b**) Graph reports the quantification of pS3-H3 positive cells 2 hours after 2 Gy, evaluated by counting IF labeled cells, in indicated MCF-7 clones. Data are expressed as fold increase over NIR cells. (**c**) Graph reports the quantification of aberrant mitoses displayed by MCF-7 clones, in NIR or 24 hours after 2 Gy. Data are expressed as fold increase over the aberrant mitoses found in MCF-7 parental cells. (**d**) Representative pictures of cells reporting the above mentioned mitotic aberrations. Cells were stained for phospho-Ser10 Histon-H3 (pS10-H3) to identify mitotic cells (pseudocolored in green), αTubulin (pseudocolored in red) to highlight the mitotic spindle and DAPI (pseudocolored in blue) to identify the cell nuclei. *Indicates a p < 0.05; **p < 0.01.

We also investigated whether the early G2 checkpoint was properly activated and the mitosis of these cells was properly executed, as previously observed in mouse fibroblasts. Following DNA damage, the cyclin B1/CDK1 complex is immediately inactivated to prevent that damaged cells enter the mitotic division^2, 29^. Kinase assay of cyclin B1-bound CDK1 indicated that p27KO cells did not display the necessary drop in kinase activity that was observed in all other p27 expressing MCF-7 cells (Fig. 6a). Differently from what observed in mouse fibroblasts, the mitotic index of MCF-7 cells, evaluated 1 hour after 2 Gy IR using the phospho-Ser10 Histone H3 marker, showed that MCF-7 p27KO clones displayed higher number of pH3 positive cells (Fig. 6b).

Finally, as observed in mouse fibroblasts, counting the number of aberrant mitoses after IR indicated that p27KO clones displayed a significantly higher number of aberrations when compared with clones expressing the p27WT protein (Fig. 6c and d).

## Discussion

The effects of p27 loss when exposed to low doses of radiation, particularly in human models, are relatively unknown. Here, we generated mouse and human cellular clones knock out for CDKN1B gene and characterized their responses to genotoxic stimuli, especially ionizing radiations.

Our results clearly indicate that expression of p27 is necessary for a proper DNA damage response. We also observed that nuclear localization and cyclin- CDK-binding ability are critical to mediate these responses. Loss of p27 was associated to accumulation of residual DNA damage, followed by increased number of mitotic aberration and genetic instability but not clonogens kill, suggesting a mechanism by which genetic unstable cells can survive cytotoxic insult.

One intriguing finding we observed is that, following IR and in the absence of p27, mouse normal fibroblasts (3T3) are still able to properly activate the G2 checkpoint; this was in contrast to human cancer cells in which G2 checkpoint activation required p27 expression. This apparently contrasting result could have at least two possible explanations. It may be due to an intrinsic species-related different sensitivity to genotoxic insults. Indeed, mouse p27 protein lacks important residues, highly conserved among primates and humans, which represent control switches for the activation of checkpoint pathways. One of them is the recently discovered S140, phosphorylated by ATM following IR and leading to protein stabilization in the nucleus^30^. The other non-conserved residue is the T157, phosphorylated by AKT following proliferative stimuli and leading to protein retention in the cytoplasm^31, 32^. Both these modifications may of course play a major role in functionally activating p27 to safeguard the genetic stability of the human cells (and thus explaining why loss of p27 has extreme consequences for human carcinogenesis). The second possible explanation relies on the different responses that can be elicited by irradiating a normal (immortalized fibroblast) or a cancer (breast cancer epithelial) cell. It is quite predictable that multiple layers of control are already lost in a cancer cell line, making the loss of p27 the final step toward the unleashing of the cell cycle and the induction of genomic instability, which is an important hallmark of cancer progression.

Normal cells have evolved intricate checkpoint responses to preserve genomic integrity in response to cellular stresses, such as the DNA damage induced by IR. Cell cycle checkpoints take crucial part to these safeguard mechanisms, in order to exclude that a DNA damaged cell progresses and, eventually, divides. As universal CDK inhibitor, p27 has been involved in the DNA damage response and recent evidence directly links p27 downstream of ATM activation for the establishment of a G1 DNA damage checkpoint arrest. However, while Cassimere *et al*. find that partial loss of p27 leads to increased susceptibility to IR^30^, we find that complete loss of p27 leads to increased survival of breast cancer cells, possibly highlighting the disruption of further control mechanisms. It is interesting to note that CDKN1B mutations found in luminal breast cancer include the K134fs, which results in the loss of p27 C-terminal domain, thus including Ser140 controlled by ATM during response to DNA damage^13^. In line with this possibility, our results clearly point to a role in G2/M checkpoint, rather than in G1/S. This is in line with our recent data, showing that p27 C-terminal portion could have an impact on the G1/S transition of the cell cycle *via* the regulation of vesicles recycling^24, 33^.

Genomic instability has potentially devastating effects and represents the hallmark of very aggressive cancers. Using highly controlled model systems, we have highlighted the importance of p27 expression to preserve genomic integrity and to efficiently recognize and clear out aberrant cells after irradiation, if the damage cannot be solved. Since radiotherapy, with hormonal therapy, represents the standard of care for most LBC patients, our study provides potentially clinically relevant insights on the mechanisms underlying radio-resistance.

**Figure 7:**
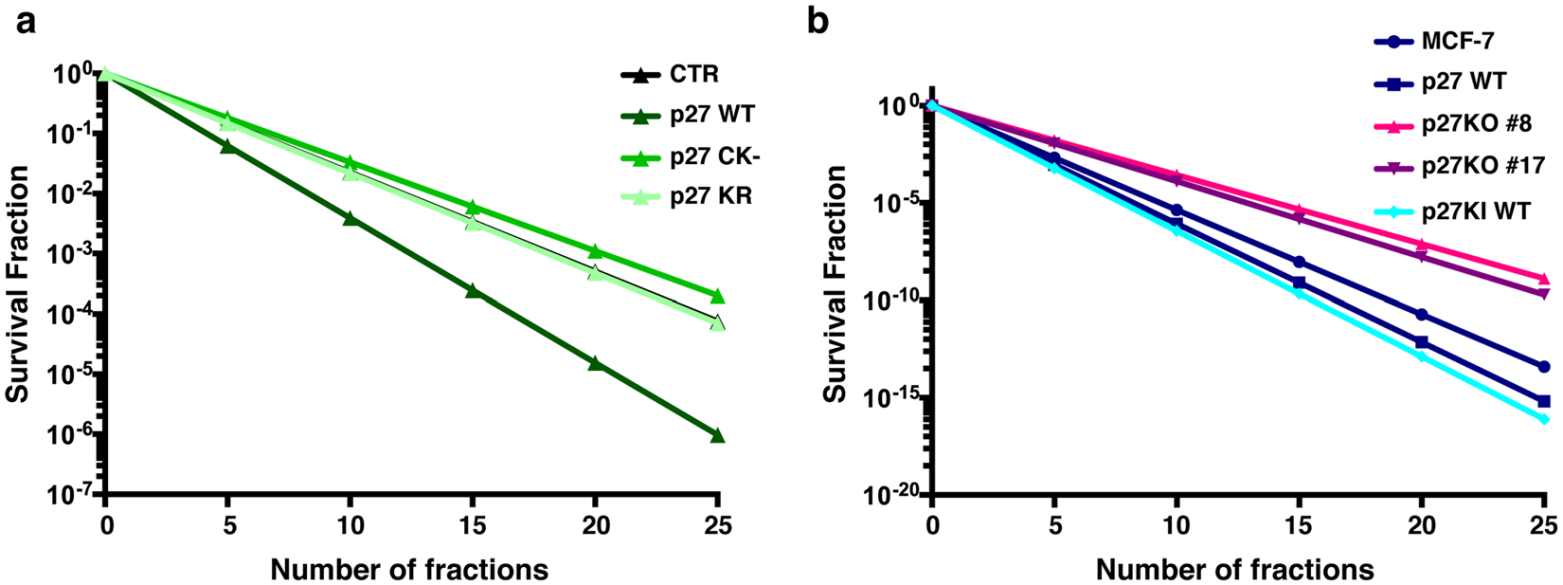
Fractionation effect on cell survival. (**a**,**b**) Graphs report the hypothetical model curves for final surviving fraction of murine 3T3 (**a**) and human MCF-7 (**b**) cell lines that are isogenic for p27. The model was calculated using the survival fraction data obtained in the cell lines after a single dose of 2 Gy to estimate the survival fraction after a 50 Gy of fractionated treatment. Final surviving fraction after 25 treatments of 2 Gy can be anywhere between two (in 3T3 fibroblasts, proficient or deficient for p27) or five logs (human MCF-7 breast cancer cells deficient or proficient in p27 expression) of cell kill difference.

Patients are treated with multiple small doses (2–3 Gy) of radiotherapy (fractionated radiotherapy) up to total doses in the range of 50 Gy. Small differences in the survival of cells following 2 Gy may therefore lead to important differences at the end of a treatment protocol assuming an equal cell kill *per* 2 Gy fraction. We modelled such an effect using the SF_2Gy_ data amongst our p27 isogenic cell clones (Fig. 7a and b). These results show that following 25 fractions of 2 Gy there can be 2–5 logs of cell kill difference in the presence and absence of p27 in both murine fibroblasts and human breast cancer cells. Given it is expected that approximately 1 × 10^9^ cells are contained within 1 gram of tumor and that 1–10% could be cancer stem cell clonogens, a difference in a final cell kill between 1 × 10^9^ versus 1 × 10^15^ would be the potential difference in tumor control (all clonogens killed) *versus* lack of control, based on p27 expression. This profound biological effect could then have crucial clinical implications and also lead to new therapeutic opportunities.

Our study demonstrates that after irradiation, loss of p27 is associated to accumulation of residual DNA damage and increased number of mitotic aberration. We show that p27 expression is necessary to preserve genomic integrity and for correct recognition and clearing-out of aberrant cells. We provide important insights into the mechanism underlying radio-resistance and unveil the possibility for novel treatment options in luminal breast cancer patients, carrying p27 mutations or in which p27 expression is lost for post-translational regulations.

## Methods

### Cell culture and Proliferation assay

Primary wild type (WT) and p27 knock out (p27KO) mouse embryo fibroblasts (MEF) were prepared from embryos at day 13.5, according to standard procedures, frozen at passage 1 and used by passage 5. 3T3 fibroblasts were obtained from primary MEFs following the 3T3 immortalization protocol, as previously described^15, 16^. 3T3 fibroblasts and MEFs were cultured in Dulbecco modified Eagle medium (DMEM) supplemented with 10% fetal bovine serum (FBS). 293T/17 (ATCC) were used for the production of retroviral particles following manufacturer’s instructions (Clontech) and cultured in DMEM supplemented with 10% FBS. MCF-7 cells were obtained from ATCC (LGC Standards) and grown in DMEM supplemented with 10% FBS. All cell lines were grown in standard conditions at 37 °C and 5% CO_2_.

For growth curves, 1 × 10^5^ cells/well (3T3 fibroblasts) or 5 × 10^4^ cells/well (MCF-7) were seeded in 6-well plates in complete medium in duplicate or triplicate. Fresh medium was added every other day. At the indicated times, cells were detached in trypsin-EDTA and counted by Trypan Blue exclusion test.

### Generation of stable cell clones

Stable cell clones of 3T3 fibroblasts were obtained by retroviral transduction (murine stem cell virus retroviral vectors, MSCV; Clontech) of particles encoding for human p27^WT^, p27^KR^ mutant (K165, R166 in Alanine) p27^CK−^ mutant (R30, L32, F62, F64 in Alanine), as previously described^16, 24^. Clones were selected in complete medium supplemented with 1.5 µg/ml of Puromycin. The stable expression of the different constructs was tested by Western Blot analysis.

Stable p27 Knock Out (KO) cell clones of MCF-7 cells were obtained by Nucleofection of custom Zinc Finger Nucleases (ZFNs) pair for p27 genomic sequence using the AMAXA V kit for electroporation (Lonza), following the manufacturer instruction. Custom ZFNs targeting the genomic CDKN1B region GGGAGCCCTAGCCTGgagcggATGGACGCCAGGCAG (lower-case letters indicate the cut site) were purchased from Sigma-Aldrich. Cells were maintained at 30 °C for 2–3 days, then shifted at 37 °C for additional 10–15 days before being single-cell seeded into 96-well plates. To confirm the ZFN activity in the clones, genomic DNA was extracted with Lysis solution for Blood and Neutralization solution for Blood (SIGMA) and CEL-I assay (Transgenomics Surveyor Nuclease Kit) performed. PCR products from CEL-I positive clones were cloned into pCR™ 2.1-TOPO® vector (Invitrogen) and sequenced to confirm deletion. Positive clones, resulted KO in at least 25 different Sanger sequences, were next analyzed by Western Blot and, finally, confirmed by Next Generation Sequencing (MySeq V2 Kit, Illumina). Two subsequent rounds of transfection and single cell cloning have been necessary in order to obtain p27KO clones in MCF-7 cells.

To obtain the MCF-7 p27 Knock In (KI) clones, the coding DNA sequence of GFP-p27 WT was cloned downstream a CMV promoter into a pZDonor-AAVS1 vector, a donor plasmid for transgene integration into the human AAVS1 locus (adeno-associated virus integration site 1), located in the human chromosome 19 (SIGMA). The donor vector (pZDonor-AAVS1-GFP-p27WT) was co-transfected by electroporation into one of the two generated p27KO MCF-7 clones, along with the mRNA coding for the ZFN specific for AAVS1 locus (SIGMA). Also in this case, cells were maintained at 30 °C for 2–3 days, and at 37 °C for an additional 10–15 days before being single-cell seeded into 96-well plates. The single-cell cloning was screened by PCR with hp27 KI primers to verify the integration of p27 coding sequence and by Western Blot analysis to verify the protein expression.

In CEL-I assay, CDKN1B regions were amplified with the following primers: hp27 ZFN-binding Fw, 5′-CGTCATTGTCTGAGTAGGTGTCA-3′; hp27 ZFN-binding Rv, 5′-GATCAACCCACCGAGCTGTT-3′. Sanger sequencing was used to confirm the mutation in CDKN1B gene, using the following primers: M13 Fw, 5′-GTAAAACGACGGCCAG-3′; M13 Rv, 5′-CAGGAAACAGCTATGAC-3′. Primers used for the MCF-7 p27 KI single-cells clonal screening were: hp27 KI Fw, 5′-CATATGTCAAACGTGCGAGTG-3′; hp27 KI Rv, 5′-AAGCTTTTACGTTTGACGTCTTCT-3′.

### Irradiation and Clonogenic Survival assay

Irradiations were performed using a ^137^Cs irradiator (Nordion) at a dose rate of ~1 Gy/minute or a Clinac 600 C (Varian Medical Systems, Palo Alto, CA) linear accelerator (LINAC) for external beam radiation therapy, at ambient oxygen concentrations and in cell adhesion conditions. In the case of irradiation with the LINAC, cell plates were positioned at the center of the radiation field of 40 × 40 cm^2^ size, with LINAC gantry at 180°, between two 5 cm layers of solid water. The dose delivered to the cell plates was 2 or 5 Gy at a dose rate of ~2.5 Gy/minute, as calculated from measurements with radiochromic films in the same setup of irradiation.

For the clonogenic assay the number of cells to seed was calculated using the following formula:

$$\begin{array}{rcl}{\rm{N}}^\circ \,{\rm{cell}} & = & {\rm{N}}^\circ \,{\rm{optimal}}\,{\rm{counting}}\,\mathrm{colonies}/\mathrm{plating}\,{\rm{efficiency}}\,{\rm{in}}\\  &  & {\rm{standard}}\,\mathrm{conditions}/\mathrm{likelihood}\,{\rm{of}}\,{\rm{predicted}}\,{\rm{survival}}{\rm{.}}\end{array}$$

Cells were seeded in 6-well plates or 60 mm dishes (two dilutions, in triplicate) and let adhere to the plates. Cells were then irradiated and maintained at 37 °C and 5% CO_2_ for 10–15 days, refreshing the medium every 3–4 days. Colonies were then fixed and stained with 1% (w/v) methylene blue in 50% ethanol or with 0.5 mg/ml crystal violet in 20% methanol. Colonies with more than approximately 50 cells were counted manually and clonogenic survival fraction (SF) was expressed as the relative plating efficiencies of the irradiated to the control cells. At least three independent experiments were performed for every condition. Where indicated, the clonogenic assays were performed after bleomycin treatment. Briefly, cells were seeded in 6-well plates (two dilutions, in triplicate) and let adhere. Then cells were treated with Bleomycin (6.25 µg/ml) for 2 hours at 37 °C and incubated 10–15 days to allow the growth of the colonies. Cells were then incubated for 10–15 days with appropriate culture medium changes every 3–4 days. Past 10–15 days, growth was blocked and the colonies stained and counted, as described above.

### Immunofluorescence

Immunofluorescence of 3T3 mouse fibroblasts was performed as previously described^24, 34^. Cells were seeded at 50–60% of confluence onto coverslips 24 hours before radiations. Cells were then irradiated with 2 Gy or sham irradiated and fixed at the indicated time points after radiations in 4% paraformaldehyde and 0.2% Triton X-100 in PBS at room temperature (RT). Following 20 min in fixative, cells were briefly rinsed in PBS, permeabilized in 0.5% NP-40 buffer and washed again 3 times. Cells were blocked using 2% BSA and 1% donkey serum in PBS buffer for at least 1 h at room temperature and incubated at 4 °C overnight with the primary antibodies in 3% BSA. Following 3 × 5 min antibody washes (0.5% BSA and 0.2% Tween-20 in PBS), coverslips were incubated with the secondary antibodies in 3% BSA, for 1 hour at room temperature in the dark. Coverslips were incubated in 0.1 μg/mL DAPI, after three more washes, and mounted onto glass slides with Vectashield (Vector Labs). Primary antibodies used were γH2AX (Epitomics #2212 or Millipore JBW301 05-636), p27 C-19 (Santa Cruz), β-Tubulin (Sigma). Secondary antibodies used were: Alexa Fluor 488 Donkey Anti-Rabbit (A21206) or Anti-Mouse (A21202); Alexa Fluor 568 Donkey Anti-Rabbit (A10042) or Anti-Mouse (A10037).

When indicated, 5-Ethynyl-2′-deoxyuridine (5-EdU) staining was performed according to manufacture’s instructions (Invitrogen). Briefly, cells were incubated with 10 μM EdU for 1 hour before fixation. The incorporated EdU was labeled using the Click-iT EdU Alexa Fluor 647 kit (Invitrogen).

MCF-7 cells were grown to sub-confluence on coverslips overnight, irradiated and fixed at indicated time after irradiation in PBS-4% paraformaldehyde for 20 min at RT, permeabilized in PBS with 0.2% Triton X-100 and blocked for at least 1 hour in PBS with 1% BSA. Incubation with anti-phospho-H3Ser10 and anti-γH2AX (Millipore) was performed overnight at 4 °C, followed by anti-rabbit-AlexaFluor488 or anti-rabbit-AlexaFluor 633 and anti-mouse-AlexaFluor 633 staining, respectively. Incubation with fluorescein isothiocyanate (FITC)-conjugated monoclonal anti-α-tubulin antibody (Sigma) was performed for 2 hours at RT in PBS-1% BSA. Antibody incubation was followed by nuclear staining with 5 μg/ml Propidium Iodide (PI) in PBS for 20 min at RT. Coverslips were mounted in Mowiol 488 (Calbiochem-Novabiochem) containing 2.5% (w/v) 1,4-diazabicyclo (2,2,2) octane (DABCO, Sigma). For nuclear staining with DAPI, coverslips were mounted in Fluoroshield™ with DAPI (Sigma). Imaging was performed with a Zeiss 140 epifluorescent microscope.

### FACS analysis

The cell cycle checkpoint analysis was performed by fluorescence-activated cell sorting (FACS) of cells stained for pS10-H3. Briefly, cells were irradiated with 2 or 10 Gy or sham irradiated and fixed in cold 80% methanol after 1 or 16 hours. Cells were washed twice in PBS, permeabilized in 0.25% Triton X-100 in PBS for 15 min in ice, washed once in PBS and blocked in 1% BSA for 30 min at RT. The cells were then resuspended in the antibody mix (AlexaFluor488-conjugated anti pS10-H3 in 1%BSA/0.25% Triton X-100) and incubated 1 hour at room temperature. After washing, cells were resuspended in PI solution (50 µg/ml propidium iodide, 100 µg/ml RNaseA, 1% BSA) followed by FACS analysis. The data were analyzed using WinMDI2.8 software.

### Preparation of protein lysates, immunoprecipitation, immunoblotting and Kinase assay

To extract total proteins, cells were scraped on ice using cold NETN buffer (50 mM TRIS-HCl pH 7.5, 150 mM NaCl, 1 mM EDTA, 1% NP40) or cold RIPA buffer (150 mM NaCl; 50 mM Tris HCl pH8; 0,1% SDS; 1% Igepal; 0,5% NP-40) plus a protease inhibitor cocktail (Complete™, Roche) and supplemented with 1 mM Na3VO4 (SIGMA), 10 mM NaF (SIGMA) and 1 mM DTT (SIGMA) and sonicated for ten seconds. Protein quantification was evaluated with BCA (Pierce) or Bradford (BIORAD) protein assay.

Immunoprecipitation (IP) experiments were performed using 0.1–0.4 mg of total lysate in HNTG buffer (20 mM HEPES, 150 mM NaCl, 10% Glycerol, 0.1% Triton X-100, protease inhibitor cocktail, 1 mM Na3VO4, 10 mM NaF and 1 mM DTT) with the specific primary antibodies, gently rocking overnight at 4 °C. A mix of protein A and protein G Sepharose 4 Fast Flow (Amersham Biosciences) was added for the last 2 hours of incubation. IPs were then washed six times in HNTG buffer and resuspended in 3 × Laemmli Sample Buffer (5 × Laemmli buffer composition: 50 mM Tris–HCl pH 6.8, 2% SDS, 10% glycerol, 0.05% bromophenol blue and 125 mM beta-mercaptoethanol). For immunoblot analysis, proteins were separated in 4–20% SDS-PAGE (Criterion Precast Gel, Biorad) or 10% SDS-PAGE and transferred to nitrocellulose membranes (GE Healthcare). Full-length membranes were cut into horizontal strips, in order to probe the membrane with multiple primary antibodies recognizing targets of different molecular weights. Membrane strips were blocked with 5% not fat dried milk (NFDM) in TBS-0.1% Tween 20 or in Odyssey Blocking Buffer (LI-COR, Biosciences) and incubated at 4 °C ON with primary antibodies.

Then, membranes were washed in TBS-0.1% Tween20 and incubated 1 hour at RT with: mouse immunoglobulin G True Blot Ultra (Rockland) secondary horseradish peroxidase (HRP)-conjugated antibody or with HRP-proteinA (Invitrogen) secondary antibody for ECL detection (Claritytm Western ECL Substrate, BioRad Laboratories) following the manufacturer’s instructions or with conjugated secondary antibodies (AlexaFluor® 680, Invitrogen; IRDye 800, Rockland; IRDye-800CW; IRDye-680LT, Licor) for infrared detection (Odyssey Infrared Detection System, LI-COR).

Primary antibodies were purchase from Santa Cruz: p27 C-19 (sc-528), CDK1 (sc-54), cyclin B1 (sc-245), cyclin A (sc-751), vinculin (sc-7649); Roche: GFP (11814460001); BD Transduction Laboratories: p27kip1 (610242), GRB2 (610112), CDK2 (610145); SIGMA: β-Tubulin (T8328).

For Kinase assay, cell lysates were immunoprecipitated using anti-CDK1 or control antibody, as described above. After 5 washes in HNTG buffer, one tenth of the IP was resuspended in kinase buffer (20 mM TrisHCl pH 6.8,10 mM MgCl2). Then, a kinase reaction solution containing the sample plus 50 μM ATP, γ-P32 ATP and 2 μg of H1- Histone as substrate in buffered solution (20 mM TrisHCl pH 6.8, 10 mM MgCl2) was prepared. The reaction was carried out at 30 °C for 30 min and then 2X Laemmli sample buffer was added. After denaturation at 95 °C for 10 minutes, proteins were loaded on a 4–20% SDS-PAGE (Criterion Precast Gel, Biorad). The gel was then dried and exposed on an autoradiographic film (GE, Amersham-Hyperfilm MP) at −80 °C and developed after different time intervals. Band quantification was performed using Image Lab™ Software (Bio-Rad).

### Statistical analyses

The computer software PRISM (version 4, GraphPad, Inc.) was used to make graphs and all statistical analyses. Unless otherwise indicated, data are expressed as mean with Standard Deviation (SD). In all experiments, differences were considered significant when p was ≤0.05, as calculated by two-tailed unpaired t-test.

### Ethical approval for animal experimentation

Animal experimentation was reviewed and approved by the CRO of Aviano Institutional Organism for Animal Wellbeing (OPBA) and by Italian Ministry of Health (authorization n°616/2015-PR, released to Dr Barbara Belletti on July 3rd 2015). All animal experiments were conducted in adherence with international and institutional committees’ ethical guidelines.

## Electronic supplementary material

Supplementary information
