# Supplementary material for: Loss of p27kip1 increases genomic instability and induces radio-resistance in luminal breast cancer cells
Source: Sci Rep. 2017 Apr 4;7:595. doi: 10.1038/s41598-017-00734-3 (PMC5429596; doi:10.1038/s41598-017-00734-3)
Supplement: Supplementary file 1 — Supplementary information [file 41598_2017_734_MOESM1_ESM.pdf]

# Supplementary Information

## Loss of p27$^{kip1}$ increases genomic instability and induces radioresistance in luminal breast cancer cells

Stefania Berton, Martina Cusan, Ilenia Segatto, Francesca Citron, Sara D’Andrea, Sara Benevol, Michele Avanzo, Alessandra Dall’Acqua, Monica Schiappacassi, Robert G. Bristow, Barbara Belletti, Gustavo Baldassarre.

## Inventory

Supplementary Information includes Supplementary Figures S1-S3, with legends.

## Supplementary Figures

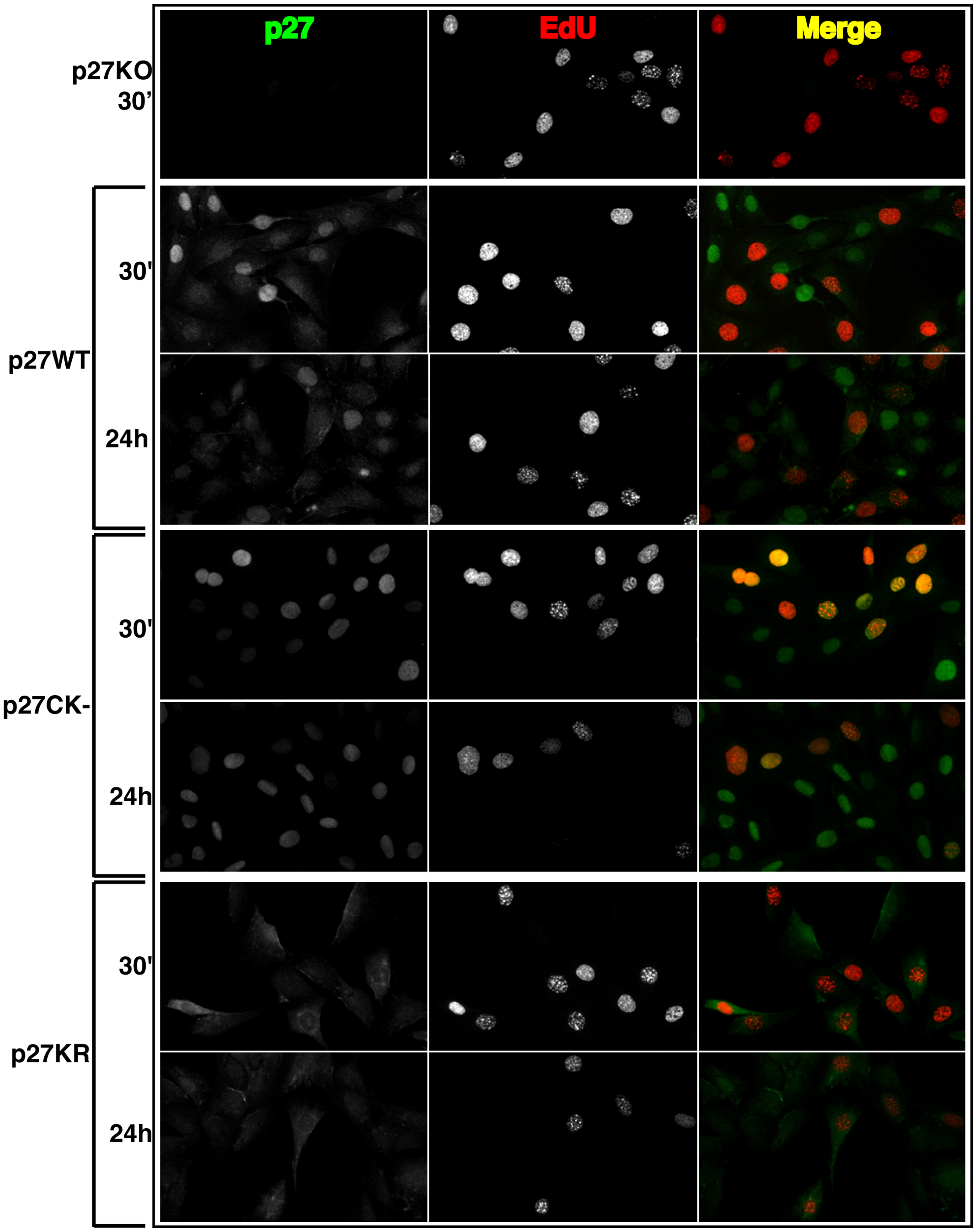


**Supplementary Fig. S1.**

**Expression and localization of p27 after IR.** p27KO fibroblasts do not express p27 and are positive for the proliferation marker 5-EdU (red). p27WT fibroblasts express p27 either in the nucleus or in the cytoplasm (green, shown in white). However, cells positive for nuclear p27 are negative for the proliferation marker 5-EdU (red, shown in white). p27CK- fibroblasts express p27 mainly in the nucleus (green, shown in

white). However, cells positive for nuclear p27 are also positive for the proliferation marker 5-EdU (yellow, in merged images). p27KR fibroblasts express p27 mainly in the cytoplasm. Cells positive for cytoplasmic p27 (green) are also positive for the proliferation marker 5-EdU in the nucleus (red). Only merged pictures on the right are shown with colors.

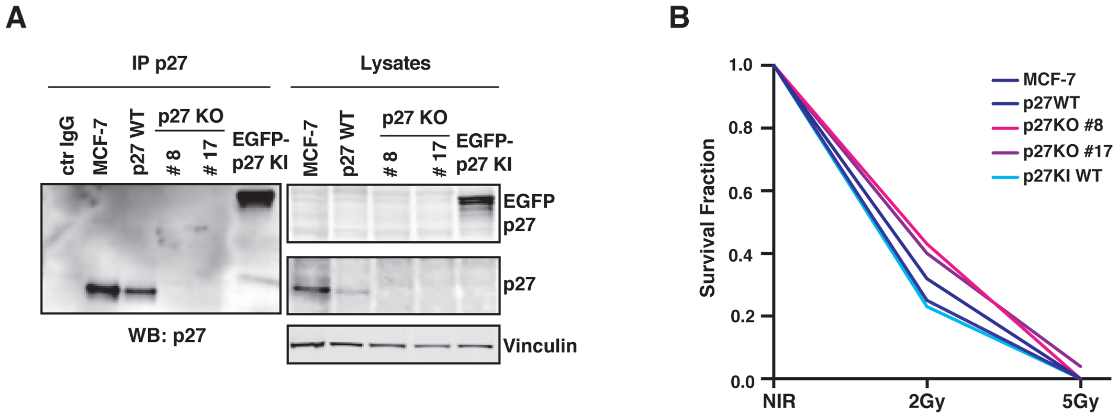


**Supplementary Fig. S2.**

**MCF-7 luminal breast cancer cells KO for p27 are more radioresistant than the parental and KI counterparts.**

**(A)** Western Blot analysis of p27 in immunoprecipitates (left panel) and in lysates (right panels), collected from the indicated cell clones and parental cell line. ctr IgG indicates a lysate immunoprecipitated with an unrelated antibody, used as a negative control. Lysates were also blotted with anti-EGFP antibody, to show the expression of the ectopic p27 in the KI clone. Vinculin was used as loading control. **(B)** Survival curve of the indicated cell clones and parental cell line irradiated with 2Gy and 5Gy and then plated in colony assay. Results are expressed as a fraction of the number of colonies counted in the non irradiated plates (NIR).

**Supplementary Fig. S3.**

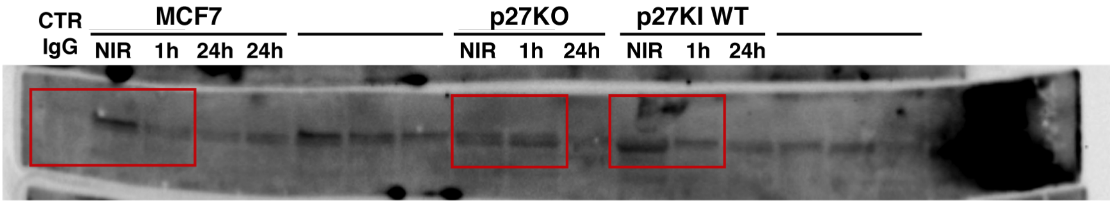


**Full length Blot of Figure 6a**

Red boxes indicate the lanes that are shown in Fig. 6a.
